# A Prospective Randomized Study on Whether Computer Navigation Is Better Than Conventional Total Knee Replacement in Terms of Short-Term Functional and Clinical Outcomes

**DOI:** 10.7759/cureus.53226

**Published:** 2024-01-30

**Authors:** Devendra Lakhotia, Utkarsh Agrawal, Shankar P Singh

**Affiliations:** 1 Department of Orthopaedics, Jaipur National University Institute for Medical Sciences and Research Centre, Jaipur, IND

**Keywords:** clinical outcome, functional outcome, conventional knee replacement, computer navigation technique, total knee replacement

## Abstract

Introduction: Computer navigation results in better radiological alignment in total knee replacement (TKR). However, functional and clinical outcomes are equally good in conventional TKR. This study aims to compare the functional and clinical outcomes in patients undergoing navigated and conventional TKR.

Methods: A prospective randomized study between navigated TKR (NKR/Group I) and conventional TKR (CKR/Group II) was carried out. Functional outcome was analyzed using the Oxford Knee Score (OKS) and Western Ontario and McMaster Universities Arthritis Index (WOMAC) score. Clinical outcome was evaluated as knee range of motion.

Results: Thirty-nine patients (68 knees) were randomized into two groups: I and II. The mean follow-up was 26 months. There was no statistically significant difference between the two groups with respect to knee range of motion, OKS, and WOMAC score at the final follow-up.

Conclusion: We concluded that there is no difference in clinical and functional outcomes between navigated and conventional TKR.

## Introduction

Total knee replacement (TKR) is one of the most important surgical advances of the 20th century. It is widely used as a surgical treatment in cases of advanced osteoarthritis and rheumatoid arthritis. It is a very successful operation assessed by pain-free knee range of motion with better functional outcomes. The longevity of the procedure depends on implant factors, patient factors, and surgical factors. The implant factors that affect the outcome of the TKR include implant size, material, and geometry. The patient factors include weight, age, presence of comorbidities, and lifestyle of the patient. The surgical factors related to implant performance include surgical skill, duration of the surgery, surgical technique, and implant position and alignment [1].

The relationship between implant malalignment and longevity has been described. For example, deviations in coronal alignment greater than 3° of varus/valgus have been correlated with poor implant survival. Shifts in the mechanical axis away from the neutral position are associated with patterns of tibiofemoral mal-tracking that lead to abnormal stresses at the bearing surface that can cause accelerated wear [2-4]. Despite the use of contemporary manual alignment systems, significant errors in mechanical axis alignment of greater than 3° are estimated to occur in at least 10% of TKR cases even performed by experienced surgeons [1]. Rotation of femoral and tibial components is particularly critical to a pain-free functional knee. Abnormal component rotation is associated with patellar mal-tracking and postoperative anterior knee pain [5,6].

Computer navigation in TKR allows accurate positioning of the component relative to the mechanical axis of the limb. It improves functional outcomes, and a better alignment results in the longevity of the prosthesis [7]. The accuracy in alignment will result in significant improvement in functional outcomes and knee scores [2,8]. Computer-navigated surgery also has decreased blood loss intraoperatively [9]. It has the advantage that the navigation system avoids instrumentation of the intramedullary canal. There is concern that intramedullary instruments may cause increased intramedullary pressure and fat embolism. This may result in respiratory symptoms with changes observed on chest radiographs, neurological symptoms, and even death [10].

On the contrary, many studies have shown that although navigation-assisted TKR results in increased implant alignment, the knee score shows no difference in functional outcome as compared to conventional TKR [11,12]. Computer-navigated TKR also has the disadvantage of more surgical time than conventional TKR which can be lengthened to 10-30 minutes [3,13,14]. Studies have also revealed that increased cost and time for navigated techniques do not translate into better function and subjective medium-term outcomes compared to conventional techniques [14].

The prolonged duration of surgery is a risk factor for an increase in surgical site infection after knee replacement [15], whereas most of the studies show a decrease in wound complication and infection after navigation TKR even after prolonged surgical time [16]. Navigation tracker pin site fractures especially on the femur side are a potential complication in navigation TKR, although correct tracker pin positioning and smaller-diameter pins could prevent this complication [17]. In view of the abovementioned facts, it is proposed to conduct a prospective study to compare clinical and functional outcomes of computer-navigated versus conventional TKR.

## Materials and methods

After obtaining approval from the Institutional Ethics Committee of Jaipur National University (approval number: JNUIMSRC/IEC/2017/34), a prospective randomized study was conducted at Jaipur National University Institute for Medical Sciences and Research Centre, Jaipur, India, on a cohort of 39 patients (68 knees) who underwent TKR surgery between May 2017 and June 2018. The study was conducted on patients of either sex undergoing unilateral or bilateral TKR. A computer-generated randomization sequence was used to randomize 68 knees in 39 patients.

Patients were divided into two groups. Group I (33 knees of 19 patients) included patients operated for TKR by computer navigation technique (NKR), and Group II (35 knees of 20 patients) consisted of patients operated for TKR by conventional instrumentation (CKR).

The inclusion criteria were patients undergoing primary TKR for osteoarthritis. In contrast, patients with inflammatory arthritis, posttraumatic secondary osteoarthritis, revision TKR, history of infective pathology, and previously operated cases for fractures or deformities were excluded from the study.

The male-to-female ratio was 5:14 in the NKR group and 5:15 in the CKR group. Out of 39 patients, five patients each in Groups I and II underwent unilateral TKR. The mean age of the cohort in Groups I and II was 63.1 years and 60.85 years, respectively, and the mean follow-up period was 26 months (range: 24-36 months). Patients' demographic data has been described in Table 1.

**Table 1:** Demographic data BMI: body mass index; TKR: total knee replacement

Variables		Group I	Group II	p-value
Number of patients		19	20	
Sex	Male	5	5	0.925
	Female	14	15	
Total knees		33	35	
Side	Right	19	20	0.971
	Left	14	15	
Laterality	Unilateral TKR	5	5	0.925
	Bilateral TKR	14	15	
Mean age (in years)		63.1	60.85	0.186
Mean BMI (in kg/m^2^)		26.8	27.6	0.524

Brainlab Knee3 (Smith & Nephew, Memphis, Tennessee, United States) imageless navigation software was used in all the knees in Group I, whereas conventional jigs were used in Group II. A single senior surgeon performed all the surgeries. Midline incision and medial parapatellar approach were used in all the cases. The implant used was NexGen (Zimmer, Warsaw, Indiana, United States) in both groups.

The preoperative evaluation of demographic factors such as age, sex, body mass index (BMI), and side of pathology was done. The difference in hemoglobin levels at preoperative and postoperative day 2 values and mean tourniquet time were compared between the two groups. The knee range of motion, Oxford Knee Score (OKS), and Western Ontario and McMaster Universities Arthritis Index (WOMAC) score were evaluated preoperatively. Postoperative evaluation was done based on patients' clinical parameters using OKS and WOMAC score at regular intervals and at the final follow-up. Preoperative and postoperative OKS and WOMAC score were compared, and a p-value of <0.05 was considered significant.

OKS is a 12-item patient-reported patient-related outcome (PRO) specifically designed and developed to assess function and pain after TKR. Each question is given numbers 1-5. The more severe the pain or loss of function, the lesser the number value is given for scoring. The maximum score is 12×5=60. Thus, a higher score means the patient has good function and less pain. WOMAC is a 24-item questionnaire including pain (five items), stiffness (two items), and physical function (17 items). The test questions are scored on a scale of 0-4. The maximum total score is 96 and the minimum score is zero. A higher WOMAC score indicates poor results in terms of pain, stiffness, and physical function [18].

Statistical analysis

Qualitative data was presented in numbers, while continuous data was presented as mean and standard deviation (SD). The normality test used was the Kolmogorov-Smirnov test. If the normality test failed, then a non-parametric test was used. The independent t-test was used to compare the quantitative data of the two groups. The paired t-test was used for comparison within groups across follow-up. The relationship between qualitative data was assessed by the chi-squared test. A p-value of <0.05 was considered statistically significant. The data was entered in an MS Excel spreadsheet, and analysis was done using IBM SPSS Statistics for Windows, Version 21.0 (Released 2012; IBM Corp., Armonk, New York, United States).

## Results

Overall, there was a significant improvement in OKS and WOMAC score in both the groups at the final follow-up. The OKS improved from 22.85 preoperatively to 57.42 postoperatively in the NKR group, while in the CKR group, it improved from 21.17 preoperatively to 58.14 postoperatively. Similarly, there was a significant change in WOMAC score from 72.85 preoperatively to 4.3 postoperatively in the NKR group and 75.43 preoperatively to 5.8 postoperatively in the CKR group. The range of motion also improved from the mean preoperative value of 99.73° to 122.88° in Group I and from 98.66° to 122.29° in Group II. However, no significant difference was noted in postoperative OKS, WOMAC score, and knee range of motion between the two groups (Table 2). Two patients in Group I had superficial infection, and one in Group II had superficial infection. None of the patients had deep infection or required revision due to any cause till the last follow-up. There was no statistically significant difference found between the two groups in age, sex, side to be operated, and BMI.

**Table 2:** Comparison of preoperative and postoperative Oxford Knee Score, WOMAC score, and knee range of motion in Groups I and II. WOMAC: Western Ontario and McMaster Universities Arthritis Index

Time period	Oxford Knee Score, mean(SD)			WOMAC score, mean(SD)			Range of motion (flexion), mean(range)		
	Group I	Group II	p-value	Group I	Group II	p-value	Group I	Group II	p-value
Pre-op	22.85 (6.82)	21.17 (3.84)	0.534	72.85 (12.96)	75.43 (7.71)	0.327	99.73 (0-110)	98.66 (0-110)	0.180
At the final follow-up	57.42 (3.22)	58.14 (3.23)	0.059	4.3 (4.87)	5.8 (3.88)	0.105	122.88 (0-130)	122.29 (0-130)	0.574

The only statistically significant differences we found in our study were the mean tourniquet time and fall in hemoglobin levels between the two groups. In the navigation group, the mean tourniquet time was 64.15 minutes, while in the conventional group, it was 48.69 minutes (p-value <0.0001). The mean fall in hemoglobin levels during the computer-navigated TKR was 1.99 gm/dl, and during the conventional TKR, it was 2.88 gm/dl (difference 0.89 gm/dl) with a p-value of <0.0001.

## Discussion

A well-aligned TKR has a higher likelihood of good functional outcomes. Studies have shown that navigation technique results in better prosthesis alignment but whether this improved alignment results in improved patient functional outcome is still unclear.

In our study, we found no significant difference in functional outcome as evaluated by OKS and WOMAC score between the two groups at the final follow-up. Similar results have been reported by Singisetti [19], Harvie et al. [20], and Kim et al. [21] in their studies with respect to functional outcomes. However, there are studies which show improved functional results of computer navigation-assisted TKR over conventional TKR. Hoffart et al. [8] reported a statistically significant better knee society score at the five-year follow-up for computer-navigated surgery. Blakeney et al. [22] found significant clinical improvement in functional scores in the navigated group in their study. There is dissimilarity in the functional outcome between the two groups in the previous studies, but the studies showing equal results outweigh the studies showing navigation as a better technique.

There are studies which suggest that radiological outcomes are better in the navigation-assisted technique but the same results are not converted into clinical and functional outcomes [7,20]. The reason for this difference might be because most of them are short- and midterm studies. Long-term studies of probably more than 15-year duration are required to establish the superiority of navigation techniques as the survival rate of TKR at 15 years is approximately 90% [23].

In our study, we found a statistically significant difference in mean tourniquet time between the two groups which was 15.46 minutes more in the navigation group (p-value 0.0001). Many studies have shown that longer surgery duration and more tourniquet time are associated with increased risks of postoperative complications like postoperative thigh pain, wound complications, nerve injuries, and higher reoperation rates [24,25]. But in most of the studies including our study, this increase in tourniquet time in the navigation technique does not increase complication risks. A study by Olivecrona et al. has shown that tourniquet time over 100 minutes increases the risk of complications after knee arthroplasty surgery [26]. The tourniquet time is more in the navigation-assisted total knee arthroplasty as evaluated in previous studies although the duration of difference is in wide range (6-15 minutes). These differences in the tourniquet time may be due to the difference in the number of surgeries at different centers and the type of navigation systems used. We assume that with high flow numbers due to an increase in navigation surgeries, more expertise, and ease of use, surgeon-friendly navigation systems in the future can cut down the tourniquet time.

Another unusual parameter that we included in our study was the mean fall in hemoglobin levels. It was observed that the mean fall in hemoglobin after two days of surgery compared to preoperative values in the computer-navigated technique was 1.99 gm/dl, while in the conventional technique, it was 2.88.gm/dl, suggesting that there is more fall in hemoglobin levels postoperatively in the conventional technique. The difference in value, i.e., 0.89 gm/dl, was found to be statistically significant (p<0.0001). Most of the studies found that blood loss is more in conventional technique although this is statistically significant in some and insignificant in others. This is also supported by the view of the previous studies showing less blood loss during the surgery in navigation TKR [9,18,27]. The reason attributed to more blood loss in conventional technique is the use of intramedullary zig for the distal femoral cuts and the opening of the femoral canal leading to increased intraoperative blood loss.

No deep infection was present in any group till the last follow-up. Only superficial infection was present in both groups but that too was insignificant. Few studies have reported less incidence of superficial infection in navigation studies [28]. Most of the studies reported equal rates of infection in both groups [29,30] as in our case series. Other intraoperative complications like fractures and thromboembolic events were not there in both groups. We did not find any case of loosening of the implant in any of our cases till the final follow-up although there is a need for a long-term study with a bigger sample size to evaluate such complications.

The limitations of our study were the small sample size and short follow-up period. The effect of navigation on prosthesis alignment and thus implant longevity is beyond the scope of this study. A study with a larger sample size and longer follow-up period is recommended to study the advantage of computer-navigated techniques over conventional techniques.

## Conclusions

We conclude that there is no difference in clinical and functional outcomes between navigation and conventional TKR. The conventional and navigation techniques both are equally good, and the technique to be used depends on the expertise of the surgeon and the financial aspect of the healthcare system.
